# Sudden cardiac death after acute myocarditis with arrhythmic presentation: hunting for risk predictors − a systematic review and meta-analysis

**DOI:** 10.1136/openhrt-2024-002985

**Published:** 2024-11-21

**Authors:** Maria Lucia Narducci, Federico Ballacci, Federica Giordano, Valentino Collini, Massimo Imazio

**Affiliations:** 1 Department of Cardiovascular Sciences, Fondazione Policlinico Universitario Agostino Gemelli IRCCS, Rome, Italy; 2 Cardiothoracic Department, Santa Maria della Misericordia University Hospital, Udine, Italy; 3 Department of Medicine, Università degli Studi di Udine, Udine, Friuli-Venezia Giulia, Italy

**Keywords:** Myocarditis, Death, Sudden, Cardiac, Defibrillators, Implantable, Arrhythmias, Cardiac, Meta-Analysis

## Abstract

**Background:**

Ventricular arrhythmias (VAs) frequently occur in the acute phase of myocarditis. Possible arrhythmic recurrences and the risk of sudden cardiac death (SCD) in this setting are reasons for concern, and limited data have been published to guide clinical management of these patients. The aim of the present paper is to report the incidence of major arrhythmic events, defined as sustained VA, SCD and appropriate implantable cardiac-defibrillator (ICD) treatment, in patients with acute myocarditis and ventricular arrhythmic phenotype.

**Methods:**

We conducted a systematic review and meta-analysis following Preferred Reporting Items for Systematic Reviews and Meta-Analyses guidelines to evaluate studies reporting long-term outcomes in patients with acute myocarditis and arrhythmic presentation. We systematically searched PubMed, EMBASE and Scopus databases for relevant studies up to 2 August 2024. Study quality was assessed by the Newcastle-Ottawa Scale. The primary outcome was a composite of SCD, VA recurrence and appropriate ICD therapy. Random-effect models were used to calculate pooled ORs and CIs.

**Results:**

Five observational studies enrolling 322 patients were identified. The pooled proportion of patients who experienced VA recurrence was 0.41 (95% CI 0.30 to 0.53, p=0.13). An increased risk of adverse outcomes during follow-up was observed in patients presenting with monomorphic ventricular tachycardia (OR 3.77, 95% CI 1.23 to 11.53) and left ventricular ejection fraction (LVEF) <50% (OR 2.74, 95% CI 0.78 to 9.63). Gender and anteroseptal late gadolinium enhancement were not found as potential risk factors in this analysis.

**Conclusions:**

Patients with myocarditis with arrhythmic ventricular presentation have a high recurrence rate of VA, underscoring the importance of careful monitoring and management in this patient population. Risk stratification for SCD during follow-up should be individualised, and monomorphic VA at presentation or a reduced LVEF may be markers of poor prognosis. In these cases, an ICD implantation may be cautious pending further dedicated studies.

**WHAT IS ALREADY KNOWN ON THIS TOPIC**

- Ventricular arrhythmias (VAs) are a known complication of acute myocarditis that requires prompt and specific management by experienced centres. Long-term risk of VA recurrence is currently uncertain in this population.

**WHAT THIS STUDY ADDS**

- This meta-analysis showed a high risk of long-term recurrence (41%) and identified presentation with monomorphic ventricular tachycardia as a marker of poor prognosis.

**HOW THIS STUDY MIGHT AFFECT RESEARCH, PRACTICE OR POLICY**

- This study highlights the importance of careful management of these patients for secondary prevention of sudden cardiac death while recognising that further research on the topic is paramount.

## Introduction

 Acute myocarditis is an inflammatory condition affecting the myocardium, presenting with a wide spectrum of clinical manifestations. These may range from a mildly symptomatic clinical picture to one characterised by chest pain, heart failure and cardiogenic shock necessitating mechanical circulatory support, as well as life-threatening ventricular arrhythmia (VA), such as sustained ventricular tachycardia (VT), ventricular fibrillation (VF) and sudden cardiac death (SCD).

It is estimated that up to 12% of SCDs in young adults may be related to myocarditis, and while a large series of 27 129 hospitalisations for myocarditis has shown that VF and cardiac arrests occur in 2.5% of patients, data on long-term arrhythmic risk prediction remain scarce.^1 2^

Current guidelines offer divergent recommendations for implantable cardioverter-defibrillator (ICD) use in myocarditis patients with malignant VA. The European Society of Cardiology suggests considering ICD for secondary prevention (class IIa, level C),^1^ while the Japanese Circulation Society recommends ICD consideration only 3–6 months after the acute phase, with potential wearable cardioverter defibrillator (WCD) use in the acute phase.^3^

Predicting the risk of adverse cardiovascular events, including sustained VA, can be a clinical conundrum for physicians, as the complex interplay between inflammatory triggers, myocardial involvement and patient-specific factors can lead to widely varying disease trajectories and outcomes.

To address these uncertainties, we conducted a systematic review of available evidence to assess the long-term risk of SCD in patients with acute myocarditis presenting with life-threatening VA.

## Methods

This study was performed in accordance with the Preferred Reporting Items for Systematic Reviews and Meta-Analyses (PRISMA) guidelines.^4^

### Search strategy

We searched for relevant articles published in PubMed, EMBASE and Scopus from inception to 2 August 2024.

The employed query included “acute myocarditis” and “myocarditis” along with “ICD”, “defibrillator” and “cardioverter-defibrillator”; “sudden death” and “sudden cardiac death”; “ventricular tachycardia”, “ventricular fibrillation” and “ventricular arrhythmia”. Reference lists of included articles were screened for additional studies.

English-language titles and abstracts were systematically screened for full paper evaluation, no other exclusion criteria were used. For multiple reports of the same data, only the most comprehensive study in relation to the review was included.

### Selection criteria

Original reports including patients with acute myocarditis and arrhythmias (aborted SCD, VT lasting >30 s, VF) during the acute phase of the disease with a minimum of 3 months of follow-up after hospital discharge were included. Reviews and case reports were excluded.

### Data extraction and quality assessment

Two investigators (FB and FG) independently reviewed the titles, abstracts and full texts for inclusion criteria. Conflicts between the reviewers were resolved by consensus. The following data were extracted from the included articles: first author, journal, publication year, country of study population and patient characteristics. For mixed populations, we reported data specific to the group of interest when available. Patient data included age, gender, major arrhythmic events (MAEs) (SCD, VF, VT) at admission and follow-up (including time to first event), ICD implantation and appropriate therapy; cardiac MRI (CMRI) data regarding the extent and localisation of late gadolinium enhancement (LGE). Study quality was assessed using the Newcastle-Ottawa Scale.

### Outcome definition

The primary outcome of interest was a composite of SCD, VF/VT recurrence and appropriate ICD therapy during follow-up. Secondary analyses focused on risk stratification based on arrhythmic presentation (sustained VT vs VF), gender, left ventricular ejection fraction (LVEF) and the presence of anteroseptal LGE on CMRI. Detailed endpoint definitions are presented in table 1.

**Table 1:** Included studies and baseline characteristics

	Rosier *et al*^7^	Gentile *et al*^5^	Sasko *et al*^6^	Cannatà *et al*^8^	Rav-Acha *et al*^9^
Study design	Multicentre retrospective	Multicentre retrospective	Multicentre retrospective	Multicentre retrospective	Multicentre retrospective
Enrolling centre location	France	Italy, France, Netherlands, Slovenia, UK, USA, Japan	Germany	UK	Israeli, USA
Population size (n)	28	156	51	18	69
Female patients (n, %)	7 (25%)	36 (23%)	14 (27.4%)	7 (38.8%)	23 (33.3%)
Age(mean±SD or median (IQR))	47±18	44 (33–55)	43 (30–56)	52.6±14	44 (36–56)
Arrhythmic presentation	sVT and VF	sVT and VF	sVT and VF	sVT, VF and complete atrioventricular block	nsVT, sVT, VF
LVEF %(mean±SD or median (IQR))	53±10	51 (42–60)	50 (45–60)	45±12	49±12
ICD implantation before discharge (n, %)	28 (100%)	93 (60%)	51 (100%)	NA	24 (34.8%)
Primary endpoint	Any appropriate ICD intervention	Composite of Sudden cardiac death; Defibrillated VF; Sustained VT requiring cardioversion or ICD therapy	Recurring arrhythmias and appropriate ICD interventions	Composite of All-cause mortality; Resuscitated cardiac arrest and appropriate ICD therapy	Composite of: All-cause mortality; Sustained VA
Follow-up duration(median (IQR or range)) in months	39.5 (21.5–81.0)	23 (7–60)	56.4 (range 12–144)	53 (34–76)	66 (10.8–144)

ICDimplantable cardioverter-defibrillatorLVEFleft ventricular ejection fractionNSnon-sustainedSsustainedVAventricular arrhythmiaVFventricular fibrillationVTventricular tachycardia

### Statistical analysis

We used a random-effects model with the empirical Bayes method to calculate the pooled risk for the primary outcome and ORs with 95% CIs for secondary binary outcomes. Sensitivity analyses employed the restricted maximum likelihood and fixed-effects models. Heterogeneity was assessed using Cochran’s Q test and I² statistic, with Galbraith plots identifying outliers when I² exceeded 50%.

Meta-regression was used to evaluate the association between logit-transformed beta-blocker usage proportion and arrhythmia recurrence risk heterogeneity. Publication bias was evaluated via funnel plots. Statistical significance was set at p<0.05 (two tailed). Analyses were performed by using Stata V.18 (StataCorp).

## Results

### Search results and baseline characteristics

**Figure 1:**
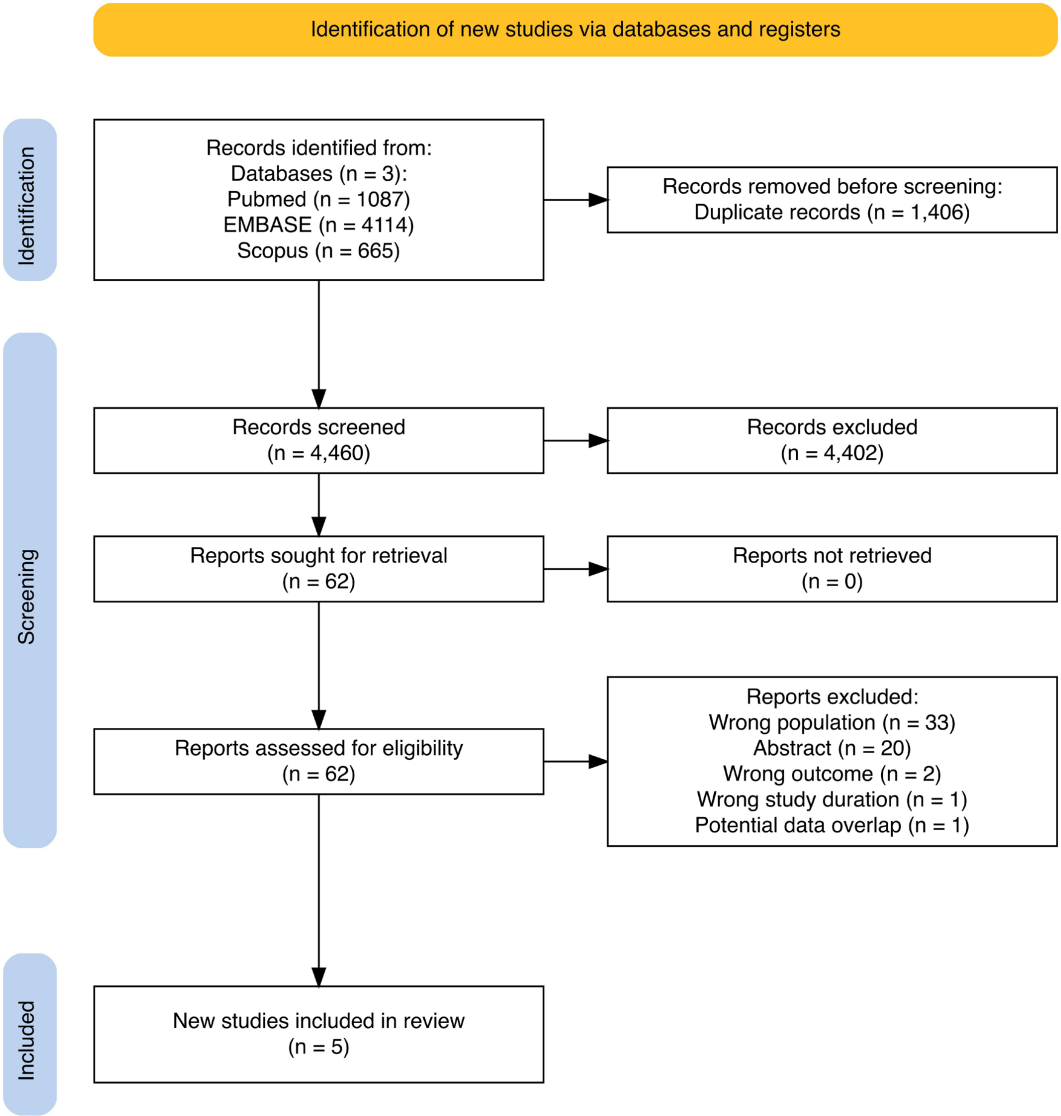
PRISMA flow diagram. PRISMA, Preferred Reporting Items for Systematic Reviews and Meta-Analyses.

A total of 5866 titles were identified, and 1406 duplicates were removed. Titles and abstracts of 4460 papers were evaluated, and 62 articles were considered for full-text screening. All 62 reports were successfully retrieved and assessed for eligibility. Of these, five studies met the inclusion criteria and were included in the meta-analysis. The references of all the included studies were searched for eligible entries, and no other study was deemed eligible for inclusion. The PRISMA flow diagram is shown in figure 1.

All studies were considered of high quality according to the Newcastle-Ottawa scale (online supplemental table S1).

A total of 322 patients (female 87, 27%) were included in the analysis. Three studies exclusively enrolled patients with VT or VF,^5^,^7^ one study also enrolled patients with complete atrioventricular block,^8^ while another study also enrolled patients with non-sustained VT.^9^

Comprehensive data on the included studies and the populations’ baseline characteristics are reported in table 1.

### Risk of cardiac death and VA during follow-up

**Figure 2:**
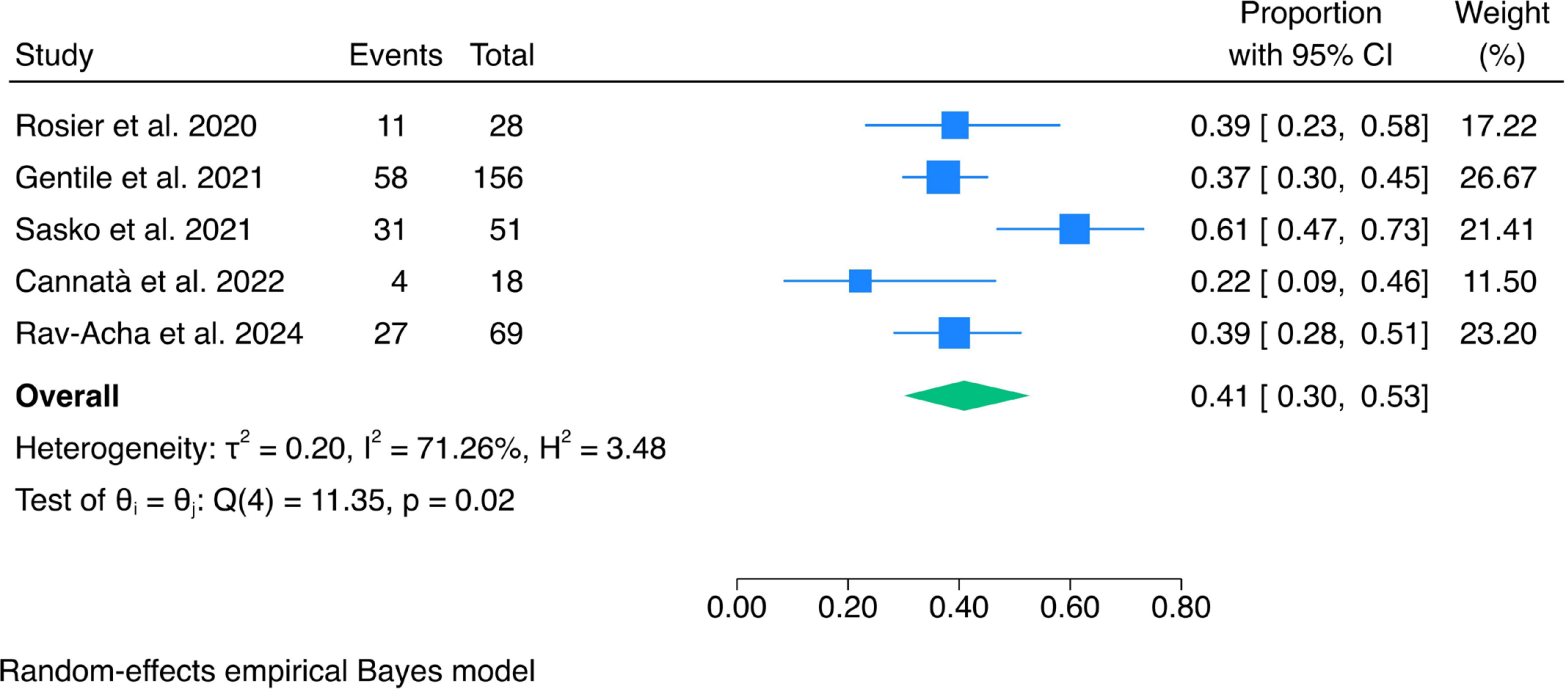
Forrest plot of risks of cardiac death and ventricular arrhythmia during follow-up.

The pooled proportion of patients who experienced VA recurrence during follow-up was 0.41 (95% CI 0.30 to 0.53, I²=71.26%) (figure 2).

Sasko *et al* ^6^ were identified as the main outlier in the Galbraith plot (online supplemental figure S1). At leave-one-out analysis (online supplemental figure S1), a reduction of the pooled risk was observed after exclusion of this study, with an estimate of 0.37 (95% CI 0.31 to 0.43). Sensitivity analyses are shown in online supplemental figure S2), and the funnel plot did not show clear evidence of publication bias (online supplemental figure S3). At meta-regression, the logit-transformed proportion of beta-blocker usage was not significantly associated with the effect size (p=0.877).

### Effect of gender on long-term prognosis

**Figure 3:**
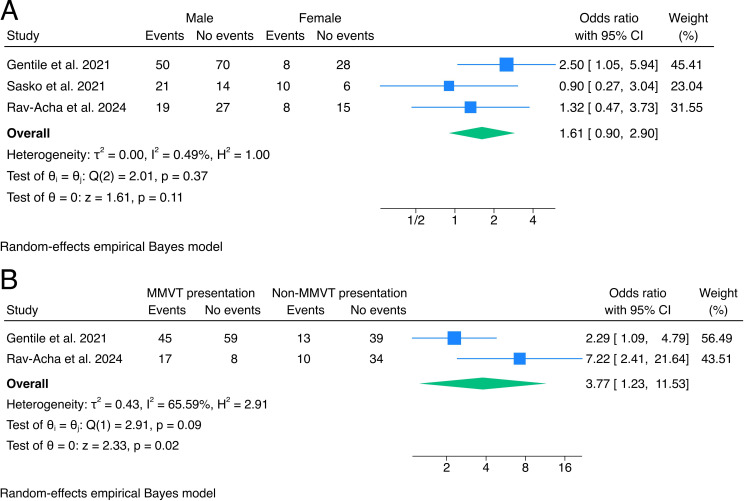
Forrest plots of the effects of gender and type of arrhythmia at presentation. MMVT, monomorphic ventricular tachycardia.

Three studies reported outcome data stratified by sex, with a pooled OR of 1.61 (95% CI 0.90 to 2.90, p=0.11, I²=0.49%) for males compared with females (figure 3A), indicating a moderate but non-significant increase in risk for males. Sensitivity analyses are shown in online supplemental figure S4.

### Type of arrhythmia at presentation

Two studies had outcome data stratified by type of arrhythmia at presentation, with a pooled OR of 3.77 (95% CI 1.23 to 11.53, p=0.02, I²=65.59) for monomorphic VT (MMVT) compared with non-MMVT presentation (figure 3B), indicating substantially increased odds of VA or SCD in patients with MMVT presentation. Sensitivity analyses are shown in online supplemental figure S5.

When considering MMVT versus polymorphic VT (PMVT)/VF, a pooled OR of 4.26 (95% CI 0.90 to 20.11, p=0.07, I²=66%) indicated a marginally significant increase in odds of VA or SCD recurrence for MMVT presentation (online supplemental figure S6). At sensitivity analyses, using a fixed Mantel-Haenszel model, the pooled OR was statistically significant (OR 3.03, 95% CI 1.56 to 5.89, p=0.001).

### LVEF at discharge of index hospitalisation

Two studies had outcome data stratified by LVEF available for the present analysis.^5 9^

**Figure 4:**
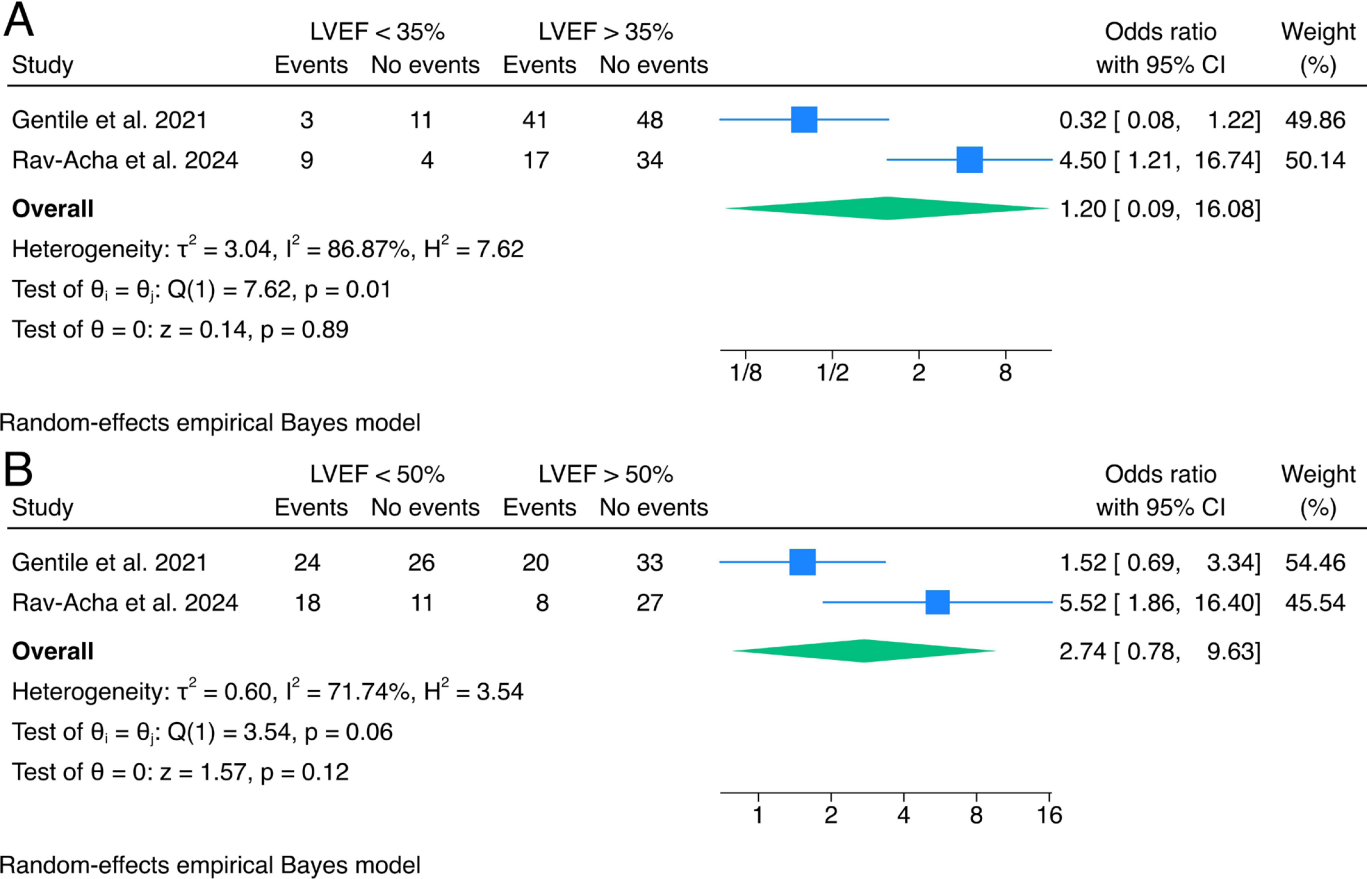
Forrest plots of the effects of LVEF during index hospitalisation. LVEF, left ventricular ejection fraction.

The pooled estimate for the effects LVEF on cardiac death and VA revealed no significant difference in outcomes between patients with LVEF cut-offs of 35% and 50%. The pooled OR was 1.20 (95% CI 0.09 to 16.08, p=0.89, I²=86.87%) for LVEF <35% (figure 4A), and individual study estimates diverged with OR 0.32 (95% CI 0.08 to 1.22) in Gentile *et al*^5^ and OR 4.50 (95% CI 1.21 to 16.74) in Rav-Acha *et al*.^9^ The fixed-effects model showed consistent results with the random-effects model.

The pooled OR for LVEF <50% was 2.74 (95% CI 0.78 to 9.63, p=0.12, I²=71.74%), and individual study estimates were OR 1.52 (95% CI 0.69 to 3.34) in Gentile *et al*^5^ and OR 5.52 (95% CI 1.86 to 16.40) in Rav-Acha *et al*^9^ (figure 4B). However, the fixed-effects model sensitivity analysis showed a pooled OR for LVEF <50% of 2.38 (95% CI 1.28 to 4.43, p=0.01), suggesting significantly increased odds of VA or SCD recurrence in patients with LVEF <50%.

### Presence of anteroseptal LGE

Two studies had outcome data stratified by the presence of anteroseptal LGE,^5 9^ the population with available data for the analysis was 78/156 (50%) of the whole population for Gentile *et al.*^5^

**Figure 5:**
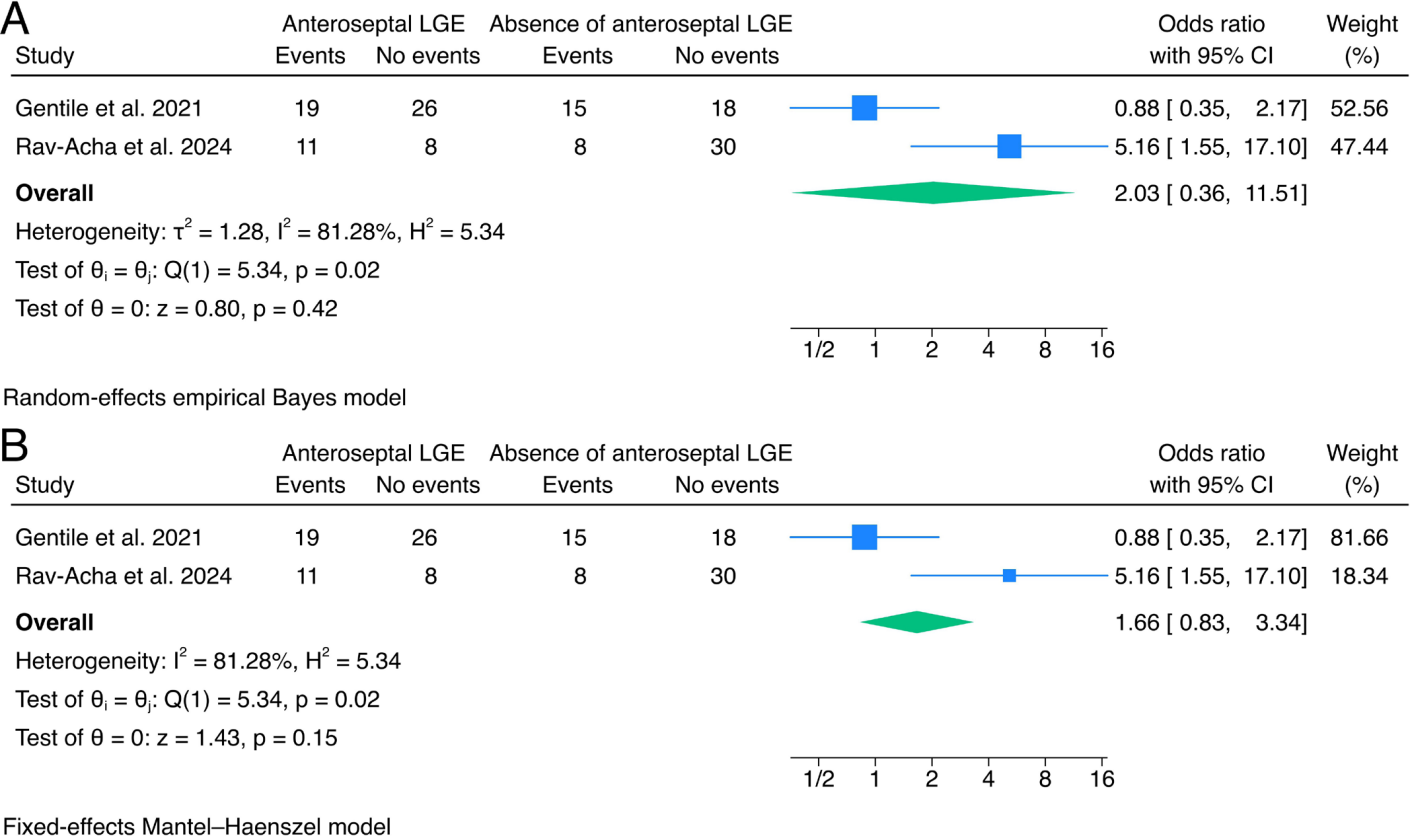
Forrest plots of the effects of anteroseptal LGE. LGE, late gadolinium enhancement.

The pooled OR 2.03 was (95% CI 0.36 to 11.51, p=0.42, I²=81.28) for the presence of anteroseptal LGE, indicating no significant differences in odds of VA or SCD recurrence (figure 5).

The two studies showed opposite directions of effect reported by Gentile *et al* (OR 0.88, 95% CI 0.35 to 2.17)^5^ and Rav-Acha *et al* (OR 5.16, 95% CI 1.55 to 17.10).^9^ The fixed-effects model showed consistent results with the main analysis.

## Discussion

Myocarditis, an inflammatory disease of the myocardium, presents with a spectrum of clinical manifestations ranging from chest pain to life-threatening VA and SCD. Grün *et al* reported that SCD was a major cause of mortality, affecting 10.9% of patients diagnosed with myocarditis,^10^ and Lynge *et al* found that 6% of identified SCD cases were diagnosed with myocarditis postmortem.^11^

Recent literature has highlighted the frequent occurrence of VA even in the acute phase of myocarditis.^57 9 12^,^14^ Approximately one-quarter of patients with acute myocarditis present with arrhythmias, with one-third of these being either VT or VF.^15 16^ Importantly, VA recurrence could be related to persistent myocardial damage after the initial episode.^17^

### Recurrence rates and current guidelines

**Figure 6:**
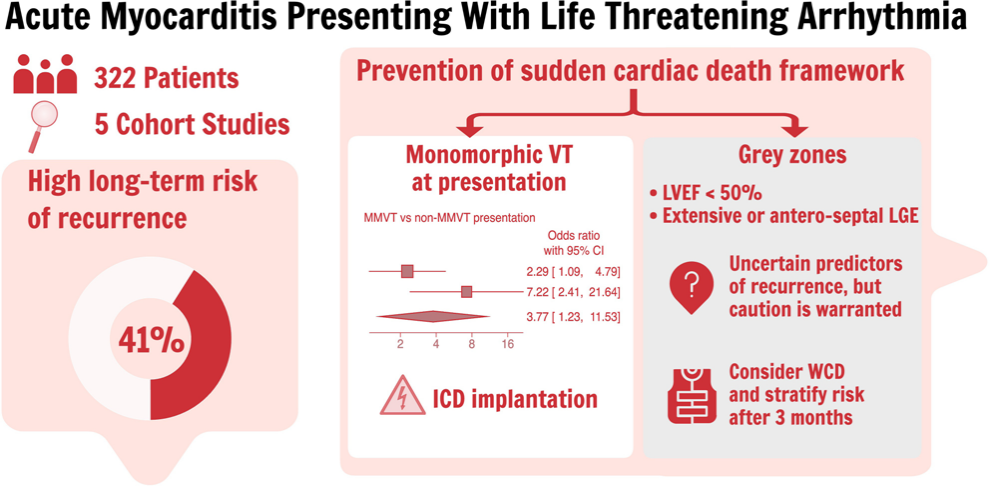
Graphical abstract: Main findings and proposed management framework for acute myocarditis presenting with life-threatening arrhythmia. ICD, implantable cardioverter-defibrillator; LGE, late gadolinium enhancement; LVEF, left ventricular ejection fraction; MMVT, monomorphic ventricular tachycardia; WCD, wearable cardioverter-defibrillator.

The reported 2-year recurrence rate of VA is high, ranging from 28% to 61%.^5 7 17^ In this regard, our meta-analysis of 5 studies, including 322 patients with acute myocarditis and VA clinical onset, revealed several important findings. The pooled proportion of patients experiencing VA recurrence during follow-up was 0.41 (95% CI 0.30 to 0.53), underscoring the importance of careful monitoring and management in this patient population (figure 6). Substantial heterogeneity was observed among the studies (I²=71.26%), primarily driven by a higher recurrence rate reported by Sasko *et al* (0.61, 95% CI 0.47 to 0.73).^6^ While some variability in reported rates is expected in relatively small observational studies, it should be noted that all patients enrolled in the study received an ICD for secondary prevention.^6^ This could imply a perceived high recurrence risk by treating physicians, as ICD implantation in this context remains controversial and often guided by clinical judgement. ICD is also a highly sensitive diagnostic tool, potentially leading to increased detection of VA recurrences. ICD implantation in the whole population was also reported by Rosier *et al*, but it should be noted that the median follow-up time reported by Sasko *et al* was also significantly longer (56.4 vs 39.6 months).^6 7^

Predictive factors include MMVT at admission, anteroseptal LGE, the absence of positive STIR sequence on CMRI and reduced LVEF. In response to these findings, the 2022 ESC guidelines recommend considering ICD implantation before hospital discharge in patients with hemodynamically unstable sustained VA during the acute phase of myocarditis.^1^ Some observational studies have also demonstrated potential benefits of WCD in this acute clinical setting.^18^

However, the level of evidence for these recommendations remains low, primarily due to the retrospective nature of available studies. The 2023 ESC guidelines on cardiomyopathy do not specifically address patients with acute myocarditis and predictors for VA recurrence, focusing on dilated cardiomyopathy and high-risk genotypes for SCD.^19^

Regarding predictors for VA recurrence in acute myocarditis, our analysis showed that gender did not significantly impact the long-term risk of cardiac death and VA (OR 1.61, 95% CI 0.90 to 2.90). However, the type of VA at clinical onset emerged as a significant predictor. Patients presenting with MMVT had substantially increased odds of adverse outcomes compared with those with non-MMVT presentations (OR 3.77, 95% CI 1.23 to 11.53).

Substantial heterogeneity was observed among the two included studies, with a significantly higher VA recurrence rate in patients with MMVT in Rav-Acha *et al*.^9^

The reported OR for MMVT presentation was 7.22 (95% CI 2.41 to 21.64), whereas Gentile *et al*^5^ reported an OR of 2.29 (95% CI 1.09 to 4.79). While Rav-Acha *et al*^9^ included NSVT in the non-MMVT presentations, the increase in OR was primarily driven by an increased risk in the MMVT group compared with Gentile *et al* (68% vs 43.3% of recurrences) rather than a lower risk in the non-MMVT group. The higher rate of VA recurrence in the MMVT group could be at least in part explained by a significantly longer median follow-up time (23 vs 66 months). Moreover, the proportion of patients with a severely impaired LVEF (<35%) reported by Rav-Acha *et al*^9^ was higher compared with Gentile *et al*^5^ (20.3% vs 13.6%).

However, patients with acute myocarditis and MMVT as clinical onset should be considered at high risk of VA recurrence, with a consequent need for a proactive management strategy with early ICD implantation. It is worth noting, however, that a thorough characterisation of the presenting VA in patients with acute myocarditis and arrhythmic phenotype is not always present in the literature, and that this result must be regarded as hypothesis-generating.

The impact of LVEF on outcomes showed mixed results. While the analysis using a 35% LVEF cut-off did not show a significant difference (OR 1.20, 95% CI 0.09 to 16.08), the analysis using a 50% cut-off suggested increased odds of adverse outcomes in patients with LVEF <50% (OR 2.74, 95% CI 0.78 to 9.63). However, it is important to note the high heterogeneity in these analyses (I²=86.87% and 71.74%, respectively). Similarly, the presence of anteroseptal LGE did not show a significant association with adverse outcomes (OR 2.03, 95% CI 0.36 to 11.51), with high heterogeneity (I²=81.28%) and studies reporting opposite directions of effect. The substantial difference in effect sizes between the studies and the relatively small sample sizes contribute to the uncertainty in the pooled estimates and should be further investigated in dedicated large prospective registries.

### Individualised decision-making

The growing body of evidence suggests the need for an individualised approach to ICD implantation in this patient population, based on two key factors: definite and accurate diagnosis of acute myocarditis based on non-invasive or invasive diagnostic tools if needed, and evaluation of ventricular arrhythmic burden, including duration and type of clinically documented VA.

Among 118 patients with a complicated myocarditis presentation (including presentation with VA in the acute phase) in the Lombardy Registry, cardiac mortality and heart transplant rate were 14.7% at 5 years, compared with 0% in patients without a complicated presentation, pointing towards an increased risk dictated by the clinical presentation.^20^ Results from the Lombardy Registry are a real-life experience of the impaired outcomes of complicated myocarditis presentations during follow-up. In particular, in this Italian registry, a complicated clinical presentation was defined in the presence of sustained VAs, LVEF below 50%, or a fulminant myocarditis. Despite the high rate of adverse events both in the short-term and long-term follow-up, the reported outcomes were not classified according to the type of clinical presentation, and consequently, we did not include these results in our analysis. It should be noted, however, that recent data challenge the notion of a benign long-term prognosis even after uncomplicated myocarditis, with a significantly higher incidence rate of cardiovascular events, including VA, compared with surgical matched controls (43.7 vs 0.9 per 1000 person-years).^21^

Other than VA at onset, several ECG could be associated with a poor prognosis in the acute myocarditis setting (pathological Q waves, prolonged QT interval, high degree AV block, QRS/T angle ≥100°)^22^; however, these markers were not consistently investigated in the studies included in our analysis.

Further investigation is needed to elucidate the roles of persistent inflammation, myocarditis relapse, and potential overlap with cardiomyopathies and associated genetic pathogenic variants in both the complicated and uncomplicated presentations of acute myocarditis.

While it is generally assumed that the proarrhythmogenic trigger dissipates after the acute phase of myocarditis, similar to postischaemic arrhythmias, recent evidence challenges this notion.

In particular, MAEs, including life-threatening VA, recur in 39% of acute myocarditis cases after resolution of the acute episode.^7^ This incidence is comparable to that observed in primary prevention ICD patients, where van Welsenes *et al* reported a 37% incidence of appropriate therapies over 5 years.^23^ In a cohort of 34 patients with myocarditis sequelae implanted with ICDs for secondary prevention, Pavlicek *et al* found that up to 58% experienced at least one VA episode within 5 years of follow-up.^24^

The persistent arrhythmic risk may be attributed not only to myocardial fibrosis, identified by LGE on CMR, but also to persistent inflammation due to ongoing autoimmune responses or viral infection.^25^ In the acute phase, viral triggers may directly damage cardiac myocytes, leading to ion channel dysfunction and interstitial oedema.^24^ Subsequent inflammation can induce conduction abnormalities, increasing the risk of fatal VA.^26^

Sasko *et al* reported a high VA recurrence rate in patients with EMB-proven acute myocarditis, and their study suggested that different viral aetiologies may contribute to varying risks of arrhythmia recurrence during follow-up.^6^ Interestingly, the absence of cytomegalovirus was associated with a lower susceptibility to VA, highlighting the potential role of specific viral agents in arrhythmia risk stratification.

In contrast, arrhythmogenesis in chronic myocarditis results from ventricular dysfunction and postinflammatory cardiac replacement with fibrotic tissue, allowing for scar-mediated re-entrant VA.^27^

Accurate disease staging is crucial for long-term VA risk stratification. Peretto *et al*^28^ demonstrated in a single-centre observational retrospective cohort study that diagnosis of active myocarditis significantly increases the risk of VT recurrence at 12-month follow-up postablation.^29^

Accurate disease staging, either through second-level imaging or EMB if needed, should therefore be performed in patients with myocarditis and ventricular arrhythmic phenotype in order to stratify the VA risk at follow-up.^30^

Early CMR assessment may provide valuable prognostic information. Gentile *et al* found that sustained VT at presentation and specific CMR patterns (LGE in >2 segments and the absence of STIR) could be key indicators for early arrhythmic risk stratification.^5^ Conversely, VF presentation with positive STIR and without extensive LGE identified lower-risk patients, potentially suitable for WCD use and follow-up CMR evaluation.

With the available evidence, the presence of LGE at first CMR does not appear to be a useful classifier to identify patients with a poor prognosis, as it is reported in the vast majority of enrolled patients in the available studies. Particularly, it should be noted that two studies considered the presence of LGE among inclusion criteria.^6 7^

Further classification of LGE is, therefore, warranted to stratify patients’ prognosis. While localisation in the anterior septum has been thoroughly described as a possible predictor of recurrence and worse outcomes in acute myocarditis with a heterogeneous presentation,^31^ pooled estimates of the available data on arrhythmic acute myocarditis do not show conclusive evidence, with specific data being scarce in the literature. Correlation of outcomes with LGE distribution (eg, subepicardial or midwall) and the pattern is a clear gap in the evidence, and it may play a role in arrhythmogenesis.

As such, the presence of extensive or anteroseptal LGE does not appear to warrant early ICD implantation but identifies a grey zone that should prompt cautious follow-up with the possibility of using wearable defibrillators as a bridge to decision.

Rav-Acha *et al* reported that 39% of patients with acute myocarditis reached a composite endpoint of sustained VA or all-cause mortality.^9^ Particularly, initial MMVT was associated with an HR of 5.17 for the composite endpoint, while predischarge LV dysfunction had an HR of 4.57. Moreover, identifying genetic backgrounds associated with high-risk profiles could improve myocarditis management.^32^ In a cohort of 336 patients with acute myocarditis, pathogenic variants for dilated cardiomyopathy or arrhythmogenic cardiomyopathy were found in 8% of patients, with a prevalence of DSP pathogenic variants in patients with normal LVEF. Genetic counselling and testing should be considered in patients with acute myocarditis and ventricular arrhythmic phenotype or reduced LVEF.^33^ In this meta-analysis, data on arrhythmic events and myocarditis relapses were not available. Dedicated studies aimed at addressing this connection are needed, in order to better identify those patients that might benefit the most from genetic testing.

### Limitations

This systematic review focusing on the available evidence in secondary prevention of SCD with ICDs in patients with acute myocarditis presenting with life-threatening VA presents several limitations. First, evidence at the acute stage of myocarditis consists of observational studies, and arrhythmic outcome was not evaluated in any randomised study to date. The retrospective nature of the included studies limits the level of evidence. Small sample sizes and the observed substantial heterogeneity may affect the reliability of the pooled estimates. In two studies, all-cause mortality was included in the composite endpoint. Although this might be regarded as a potential limitation, it is the single available proxy for those arrhythmic events that cannot be ascertained by consulting implanted device tracings. Moreover, the lack of characterisation of the study population for the secondary outcomes in some studies made it necessary to exclude a significant proportion of patients from pooled OR analyses.

Despite these limitations, this meta-analysis provides valuable insights into the long-term arrhythmic risk of acute myocarditis presenting with VA. Moreover, this meta-analysis highlights the need for dedicated prospective studies on the subject, to better weigh the relative roles of aetiology and persistent inflammation in determining long-term risk of relapsing arrhythmias.

## Conclusion

Management of VA in acute myocarditis requires a multistep evaluation approach. In the acute phase, early diagnosis of disease staging and VA characterisation are essential to effectively manage these patients in the appropriate care setting. Patients at high risk of recurrence and SCD during follow-up may be identified by careful assessment of established risk factors such as LVEF and LGE, but MMVT at presentation should be considered as a potential marker of poor prognosis. In such cases, early ICD implantation may be cautious, while dedicated prospective studies are needed to identify which patients would benefit most from early ICD implantation and to establish optimal long-term management strategies in this population.

## supplementary material

10.1136/openhrt-2024-002985 online supplemental file 1

## Data Availability

Data are available on reasonable request.
