# Supplementary material for: Sudden cardiac death after acute myocarditis with arrhythmic presentation: hunting for risk predictors − a systematic review and meta-analysis
Source: Open Heart. 2024 Nov 21;11(2):e002985. doi: 10.1136/openhrt-2024-002985 (PMC11603706; doi:10.1136/openhrt-2024-002985)

Supplemental Material.

Supplementary Table S1. Newcastle-Ottawa Scale to assess the quality of the included studies

Study label	***Selection***	***Comparability***	***Outcome***
Rav-Acha et al. 2024	4	2	3
Gentile et al. 2021	4	2	3
Sasko et al. 2021	4	2	3
Cannatà et al. 2022	4	2	3*
Rosier et al. 2020	4	2	3*

*While these studies did control for important variables, the population was not stratified in accordance with the meta-analysis methods and stratified results could not be used for quantitative analysis

Supplementary Figure S1. Galbraith plot and leave-one-out analysis for the main outcome of interest. CI, confidence intervals.

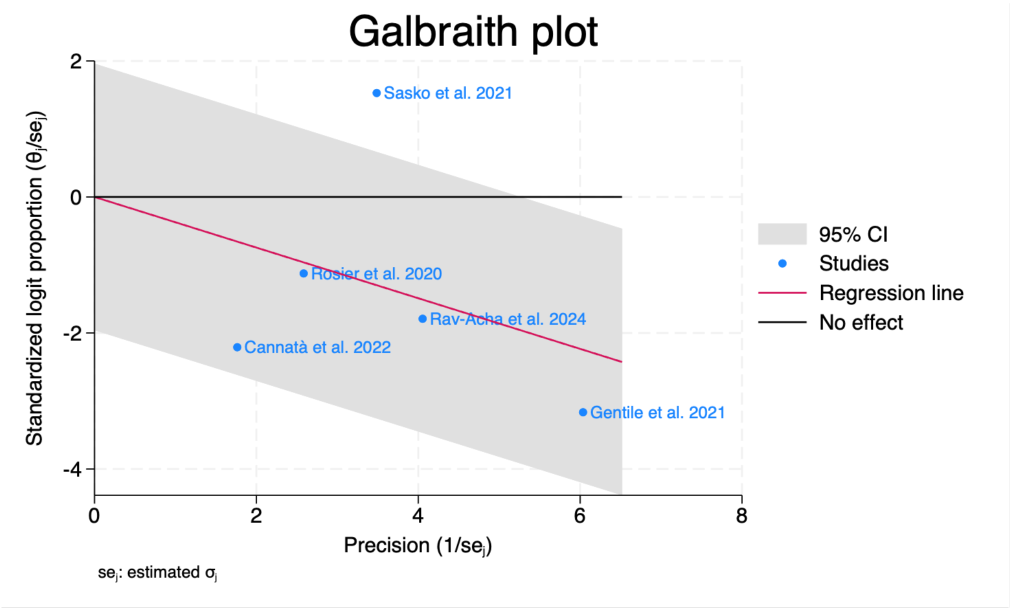

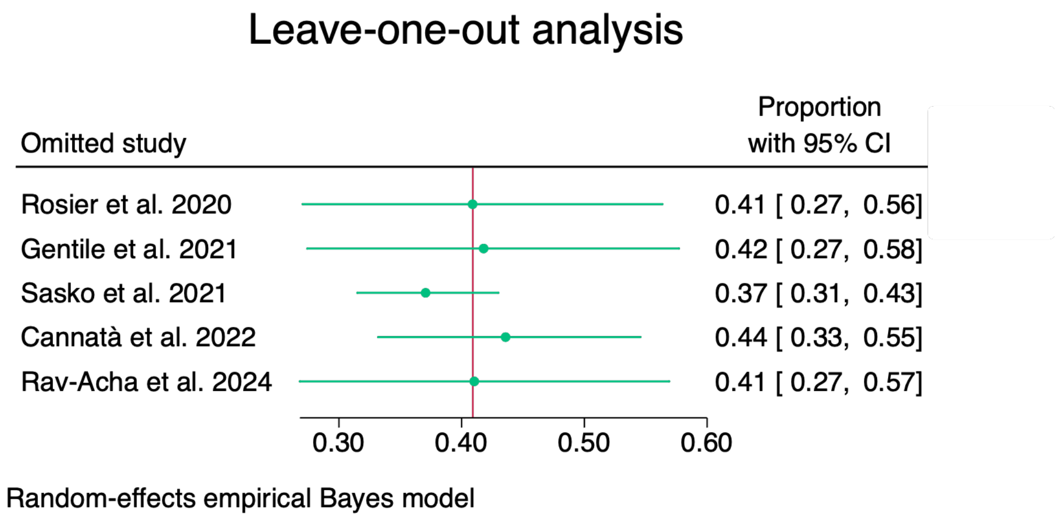

Supplementary Figure S2. Risk of Cardiac Death and Ventricular Arrhythmia during follow-up: sensitivity analyses. CI, confidence intervals.

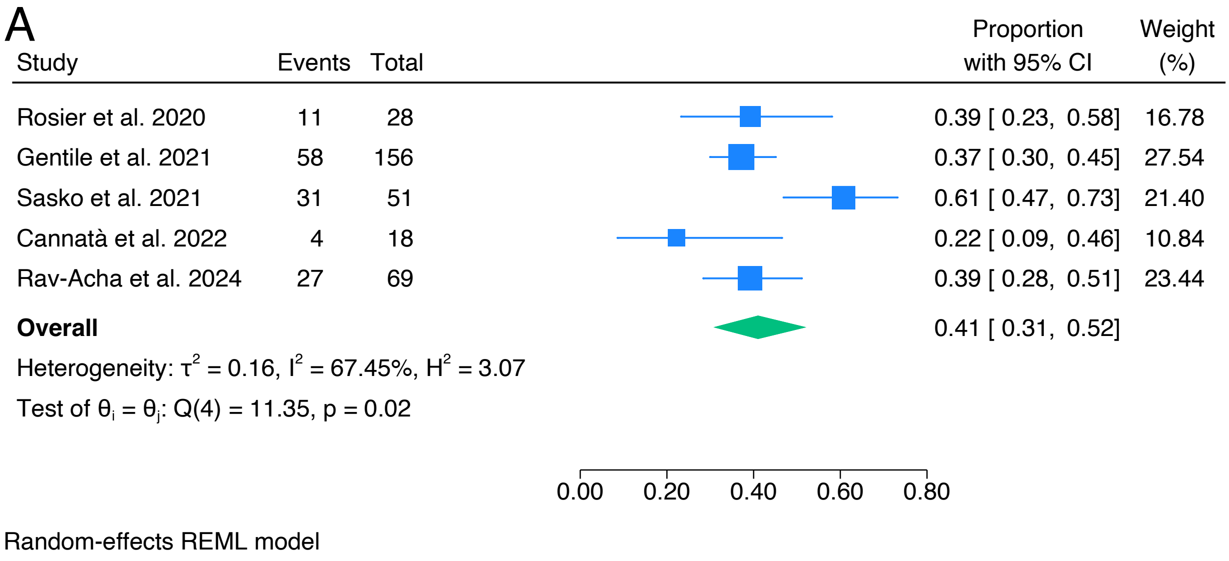

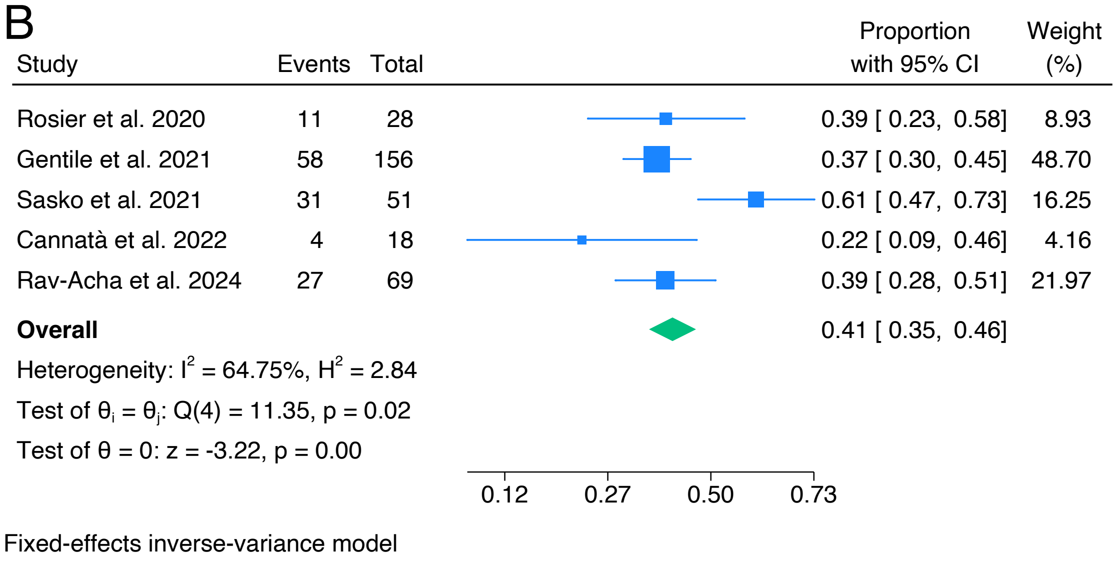

Supplementary Figure S3. Risk of Cardiac Death and Ventricular Arrhythmia during follow-up: Funnel Plot. CI, confidence intervals.

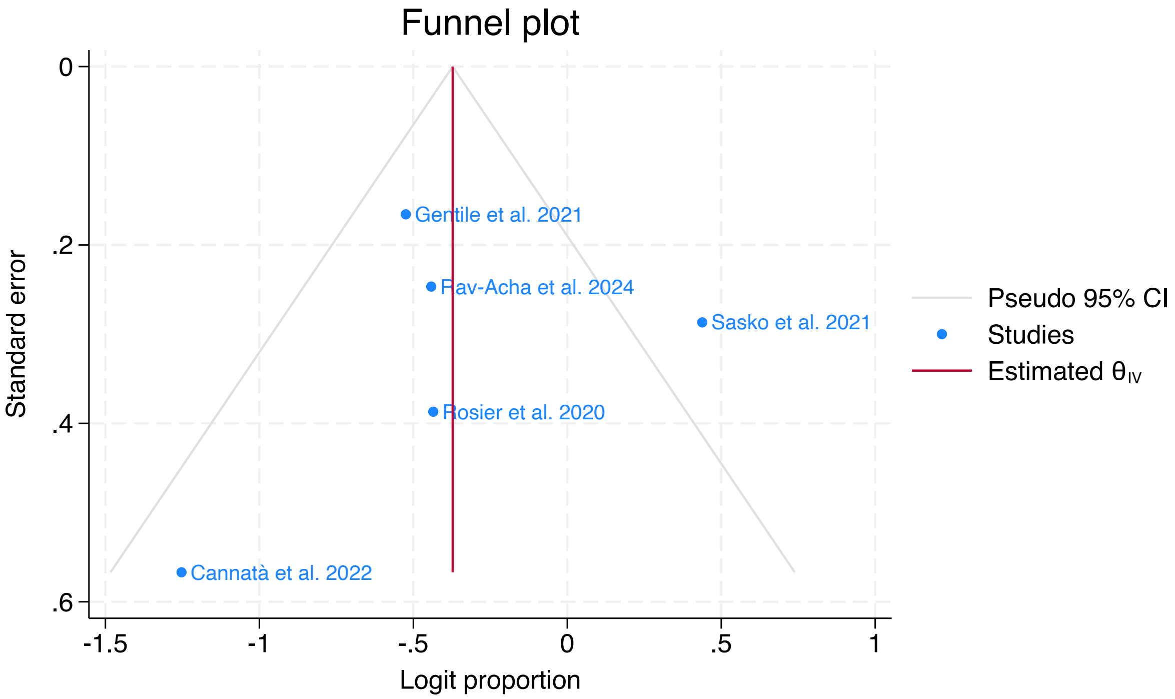

Supplementary Figure S4. Effects of gender: sensitivity analyses. CI, confidence intervals.

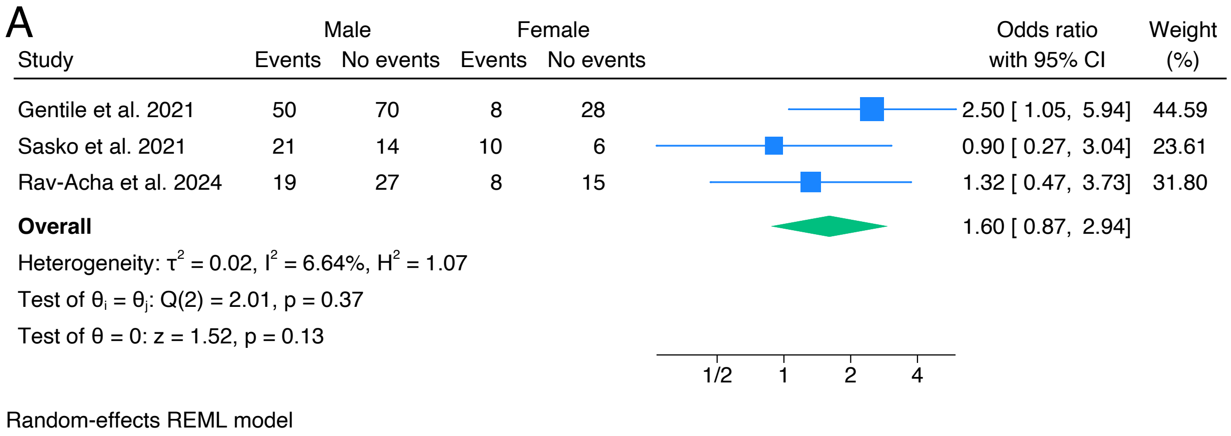

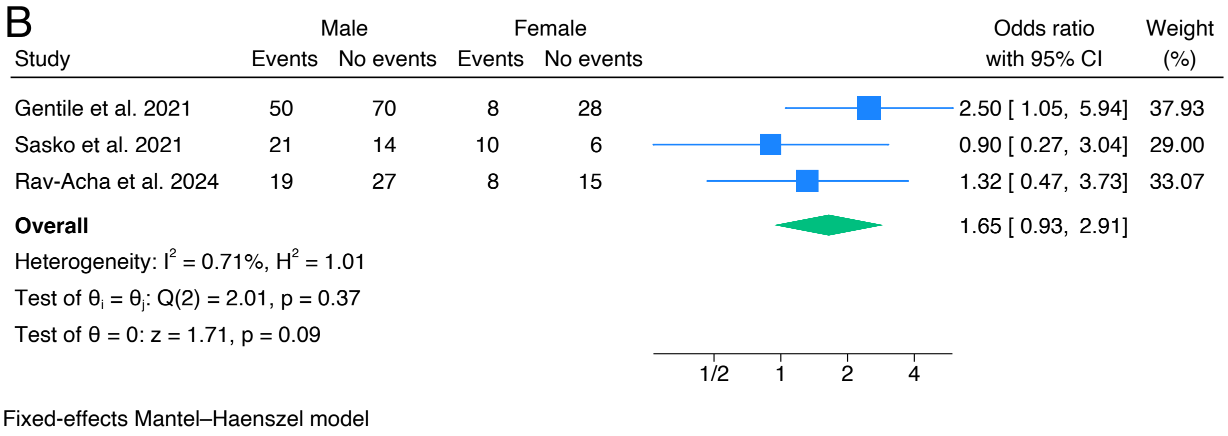

Supplementary Figure S5. Effect of arrhythmia at presentation: MMVT vs non-MMVT sensitivity analyses. MMVT, monomorphic ventricular tachycardia; CI, confidence intervals.

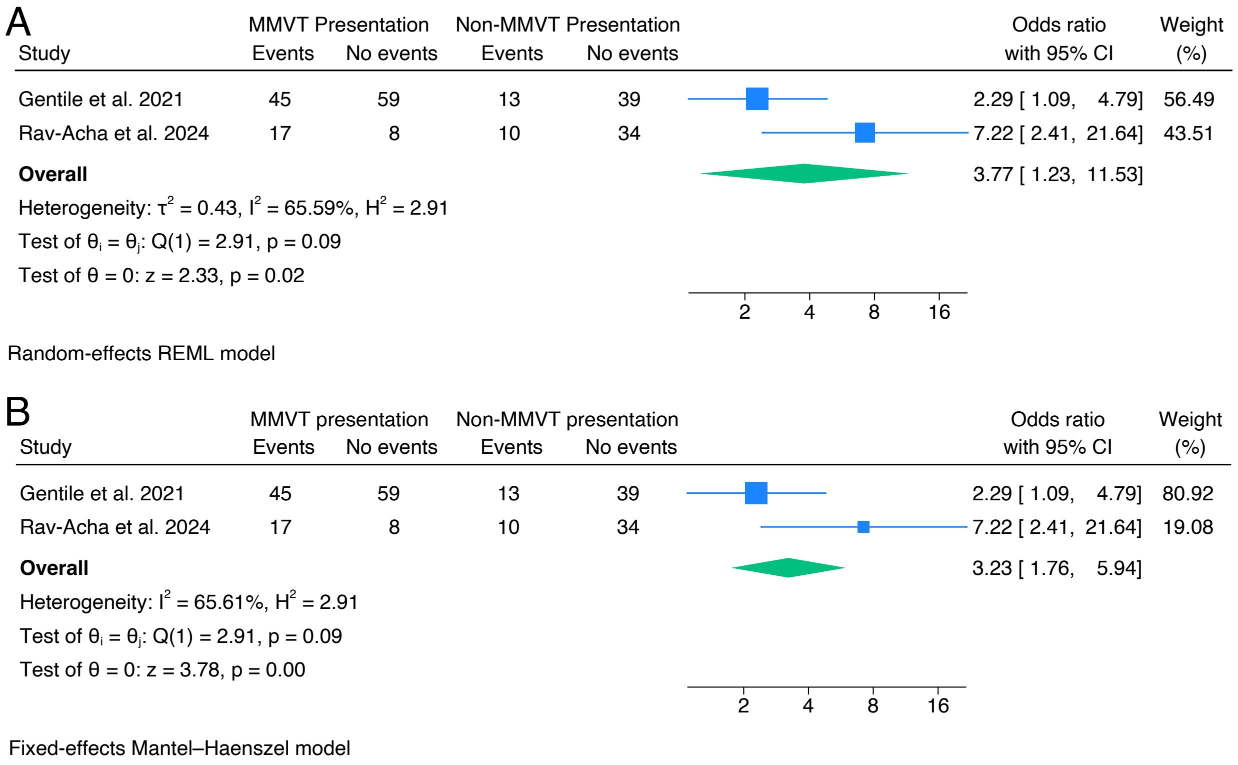

Supplementary Figure S6. Effect of arrhythmia at presentation: MMVT vs PMVT/VF. MMVT, monomorphic ventricular tachycardia; PMVT/VF, polymorphic ventricular tachycardia / ventricular fibrillation; CI, confidence intervals.

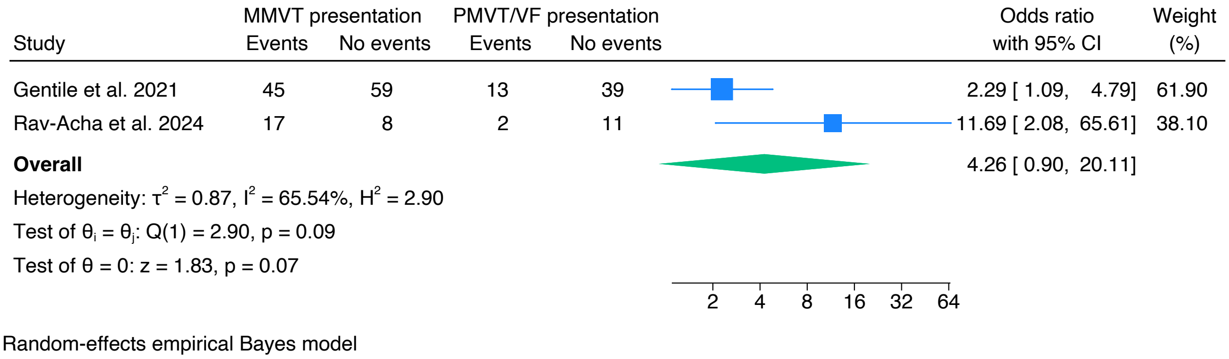

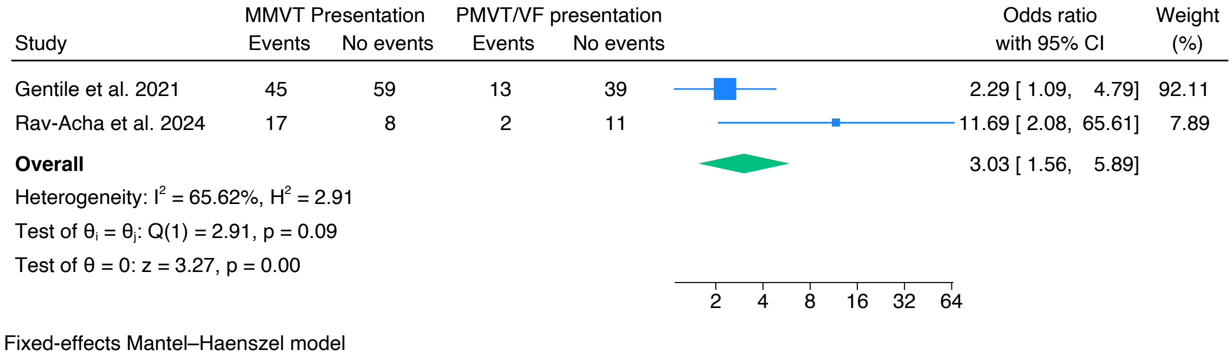

Supplement: online supplemental file 1 [file openhrt-11-2-s001.pdf]
